# The expression profiles and prognostic values of HSP70s in hepatocellular carcinoma

**DOI:** 10.1186/s12935-021-01987-9

**Published:** 2021-05-31

**Authors:** Ben Wang, Tian Lan, Han Xiao, Zhong-Huo Chen, Chao Wei, Lei-Feng Chen, Jia-Fu Guan, Rong-Fa Yuan, Xin Yu, Zhi-Gang Hu, Hua-Jun Wu, Zhi Dai, Kai Wang

**Affiliations:** 1 Hepato-Biliary-Pancreatic Surgery Division, Department of General Surgery, The Second Affiliated Hospital of Nanchang University, No. 1, Minde Road, Nanchang, 330006 China; 2 Jiangxi Province Key Laboratory of Molecular Medicine, Nanchang, 330006 China; 3 Jiangxi Province Engineering Research Center of Hepatobiliary Disease, Nanchang, 330006 China; 4 Liver Cancer Institute, Zhongshan Hospital, Fudan University, Shanghai, China; 5 Department of Health Care Management and Medical Education, The School of Military Preventive Medicine, Fourth Military Medical University, Xi’an, 710032 China; 6 Department of Health Care Management, The Second Affiliated Hospital of Nanchang University, Nanchang, 330006 China

**Keywords:** HSP70 family, Hepatocellular carcinoma, Bioinformatics, HSPA4, HSPA14

## Abstract

**Background:**

The HSP70 family of heat shock protein plays a critical role in protein synthesis and transport to maintain protein homeostasis. Several studies have indicated that HSP70s are related to the development and occurrence of various cancers.

**Methods:**

The relationship between the overall survival rate of hepatocellular carcinoma patients and the expression of 14 HSP70s from multiple databases, such as TCGA, ONCOMINE, cBioPortal was investigated. Western Blot and PCR were used to evaluate HSPA4 and HSPA14 expressions in various HCC cells to identify suitable cell lines for further experiments .Wound-healing assays, Transwell assays and EdU assays were used to verify the effects of HSPA4 and HSPA14 on the function of hepatocellular carcinoma cells, and statistical analysis was performed.

**Results:**

Hepatocellular carcinoma tissues significantly expressed the 14 HSP70s compared to the normal samples. Besides, the high HSPA1A, HSPA1B, HSPA4, HSPA5, HSPA8, HSPA13, and HSPA14 expressions were inversely associated with the overall survival rate of patients, tumor grade, and cancer stage. A PPI regulatory network was constructed using the 14 HSP70s proteins with HSPA5 and HSPA8 at the network center. Univariate and multivariate analyses showed that HSPA4 and HSPA14 could be independent risk factors for the prognosis of hepatocellular carcinoma patients. Cell experiments have also confirmed that reducing HSPA4 and HSPA14 expressions can inhibit the invasion, metastasis, and proliferation of hepatocellular carcinoma cells.

**Conclusions:**

Therefore, the HSP70s significantly influence the occurrence and development of hepatocellular carcinoma. For instance, HSPA4 and HSPA14 can be novel therapeutic targets and prognostic biomarkers for hepatocellular carcinoma.

**Supplementary Information:**

The online version contains supplementary material available at 10.1186/s12935-021-01987-9.

## Introduction

Hepatocellular carcinoma (HCC) is a common and deadly cancer. About 800,000 new cases and over 700,000 HCC-associated deaths are reported yearly [1]. Surgical treatment, including hepatectomy and liver transplantation (LT), is the main HCC treatment. Moreover, sorafenib is the only systematic therapy for HCC [2]. Despite significant progress in hepatocellular carcinoma treatment in the past, Its prognosis remains poor odue to its high invasiveness and metastasis rate. Moreover, the molecular characteristics of HCC are unknown. Understanding the potential pathogenesis and etiology of HCC can help find advanced biomarkers for HCC treatment and diagnosis, thus improving the prognosis and individualized patient treatment.

Increased conformational unstable proteins cause continuous proliferation and genetic instability of cancer cells. Also, excessive protein synthesis and metabolism lead to a stress state in cancer cells that must be permanently compensated. Therefore, tumor cells depend on the maintenance of protein homeostasis via the molecular chaperones. HSP70s are ubiquitous molecular chaperones essential for folding and remodeling of all cellular proteins. HSP70s help misfolded proteins to fold and refold correctly. HSP70s are essential in all protein synthesis and degradation stages, thus important in maintaining protein homeostasis [3]. Cancer cells can release HSP70s, which influence malignant characteristics via receptor-mediated signal transduction [4]. Therefore, HSP70s can influence HCC occurrence and development via several mechanisms. Some studies have revealed that the HSP70 expression is upregulated in some human malignant tumors, such as liver, breast, pancreatic, colorectal, and prostate cancers [5–9]. Therefore, a comprehensive study of various HSP70s in HCC is needed to clarify the molecular mechanisms involved in HCC development, providing potential therapeutic targets and prognostic markers of HCC.

Presently, 14 HSP70s members have been identified (HSPA1A, HSPA1B, HSPA1L, HSPA2, HSPA4, HSPA4L, HSPA5, HSPA6, HSPA8, HSPA9, HSPA12B, HSPA12A, HSPA13 and HSPA14). HSP70s have critical roles in tumor cells [10, 11]. Some studies have found abnormal expression of some HSP70s and their prognostic value. For instance, HSPA1A promotes hepatoma cell proliferation through TLR2 and TLR4 signaling pathways[12], and HSPA1L/HIF-1 α/GP78 significantly influences tumor progression [13]. HSPA1B (HSP70-2) down-regulation can reduce cell proliferation and tumor growth [14]. HSPA2 overexpression is associated with tumor angiogenesis, invasive progression, and poor prognosis [15, 16]. Previous studies showed that HSPA5 (GRP78) increases FAT10 expression via NF-κB pathway to promote HCC proliferation [17]. HSPA5 also accelerates HCC invasion and metastasis by activating the Wnt pathway via the LRP6 [18]. Similarly, HSPA4 [19], HSPA8 [20, 21], HSPA9 [22, 23], and HSPA12B [24, 25] are associated with tumor occurrence and development. However, the role and relationship of various HSP70s in HCC occurrence and development are unknown. Several DNA and RNA data have been produced due to the continuous progress in microarray technology. Several gene expressions or copy number variations published in the online database can be analyzed to test whether the expression of various HSP70 proteins is related to clinical parameters, such as the OS of HCC patients, and explore the function of HSP70s in HCC occurrence and development.

## Materials and methods

### ONCOMINE database

ONCOMINE database (www.oncomine.org) is a comprehensive online cancer microarray database for DNA or RNA sequence analysis that facilitates discovery from the gene-wide expression analyses [26]. Transcriptional expressions of 14 HSP70s in various cancer tissues and their corresponding adjacent normal control samples were obtained from ONCOMINE database.

### UALCAN

UALCAN (http://ualcan.path.uab.edu) is an interactive web resource based on level 3 RNA-seq and clinical data of 31 cancer types from the TCGA database [27]. UALCAN was used to analyze the mRNA expressions of 14 HSPA70s in HCC tissues and their relationship with clinicopathologic parameters. Students’ t-test was used to compare the transcriptional expression difference. P < 0.01 was considered statistically significant.

### cBioPortal

cBioPortal (www.cbioportal.org) is an online open-access website resource. It is used to explore, visualize, and analyze multidimensional cancer genomics data [28]. The genomic profiles of 14 HSP70s, including mutations, putative copy-number alterations from GISTIC, and mRNA Expression z-Scores (RNASeq V2 RSEM) with a z-score threshold ± 1.8 were analyzed.

### The Cancer Genome Atlas (TCGA) database

TCGA [29] is a coordinated and comprehensive tool that ameliorates diagnostic methods and treatment criteria for cancer prevention. Over 30 human tumor sequencing and pathological data can be analyzed on TCGA. Transcriptional group RNA sequencing data of HCC samples, including the transcriptional group data and corresponding clinical information of 377 HCC patients, were downloaded from the TCGA (https://cancergenome.nih.gov/). A total of 89 patients with less than three months overall survival, or inadequate clinical information, or no R0 surgery in the TCGA database were not excluded to avoid the impact of non-related confounding factors. The data of the remaining 288 patients were used for univariate and multivariate analyses. The results are shown in Table 1.

**Table 1:** Basic characteristics of 288 HCC patients

Variables	HCC patient number
Overall Survival Status (Alive/Dead)	194/94
Age (≤ 65/>65)	187/101
Gender (Female/Male)	96/192
Tumor Status (With Tumor/Tumor free)	90/182
Pharmaceutical Therapy (No/Yes)	190/9
Ablation embolization (No/Yes)	189/12
Neoplasm Histologic Grade (I/II/III/IV)	42/135/96/11
T Stage (1/2/3/4)	145/72/60/8
N Stage (N0/N1-NX)	207/80
M Stage (M0/MI-MX)	217/71
Neoplasm Disease Stage (I/II/III/IV)	138/67/64/2
Vascular Invasion (None/Micro/Macro)	161/72/13
Child pugh classification grade (A/B/C)	182/20/1
Adjacent hepatic tissue inflammation extent type (None/Mild/Severe)	102/85/14

### Functional enrichment analysis

STRING v11 database (https://string-db.org/) was used to identify 30 frequently neighbor genes of HSP70s. DAVID database (https://david.ncifcrf.gov/summary.jsp) was then used to analyze the function of HSP70s and the 30 genes significantly related to HSP70s, such as cellular components (CC), biological processes (BP), molecular functions (MF), and KEGG analysis.

### Cell culture and transfection

HCC human cell lines, including HCCLM3, Huh-7, SMCC7721, and MHCC97H, were sourced from the Institute of Biochemistry and Cell Biology, Chinese Academy of Sciences (Shanghai, China). Cell Bank confirmed the cells using short tandem repeat profiling. The Cells were cultured as previously described. Lipofectamine™ 3000 Transfection Reagent (Invitrogen, Carlsbad, USA) was used for transfection [17].

### Western blot analysis and Real-time quantitative polymerase chain reaction (qRT-PCR)

Western blotting and qRT-PCR were performed as earlier mentioned [30]. The specific primers used for PCR amplification are shown in Additional file 1: Table S1.

### Wound-healing assays

Use the marker pen to draw a horizontal line evenly on the back of the 6-well plate, about every 0.5–1 cm, across the well. The cells were cultured in 6-well plates to 80–90%. Scratched the horizontal line perpendicular to the back with the pipette tip. Washed the cells gently with PBS for 3 times and added serum-free medium. Continue culturing in incubator at 37 ℃. Observed and took pictures at the time point of 0 h, 24 h, 48 h [30].

### Transwell assays

5 × 10^4^ cells were suspended in serum-free medium and placed in the upper chamber. After 24 h of culture, the unmigrated cells on the upper surface of the membrane were carefully removed with cotton swabs, the cells on the lower surface of the membrane were fixed and stained with 0.1% crystal violet, observed and photographed [18].

### EdU assay

The cells were treated with 150 µl 5-ethynyl-20-deoxyuridine (EdU) (Ribobio, Guangzhou, China) for 2 h. Washed the cell with PBS for three times. After cells were treated with 2 mg/ml glycine and 0.5 % TritionX-100 in turn,the cells were stained with 300 µl of 1×Apollo® reaction cocktail for 30 min. Then, the cells were stained with 300 µl of Hoechst 33,342 (5 µg/ml) for 30 min. Washed the cells with PBS after each step. Observed and took pcitures  [31].

### Statistical methods

R v3.6.1(https://www.r-project.org/) and SPSS 26.0(SPSS Inc., Chicago, IL) were used for all statistical analyses. Univariate Cox algorithm was used to analyze and evaluate the effects of clinical parameters and HSP70s mRNA expression on the survival of HCC patients. Those with P < 0.1 were used for follow-up analysis. A Multivariate Cox algorithm was used to analyze the relationship between HSP70s mRNA expression and patient survival. P < 0.05 was considered statistically significant. Each experiment was repeated thrice. All values were expressed as an average of ± SD. T-test was used to analyze all experimental results, * P < 0.05,** P < 0.01 and *** P < 0.001.

## Results

### HSP70 expressions in the HCC and normal tissues

**Fig. 1:**
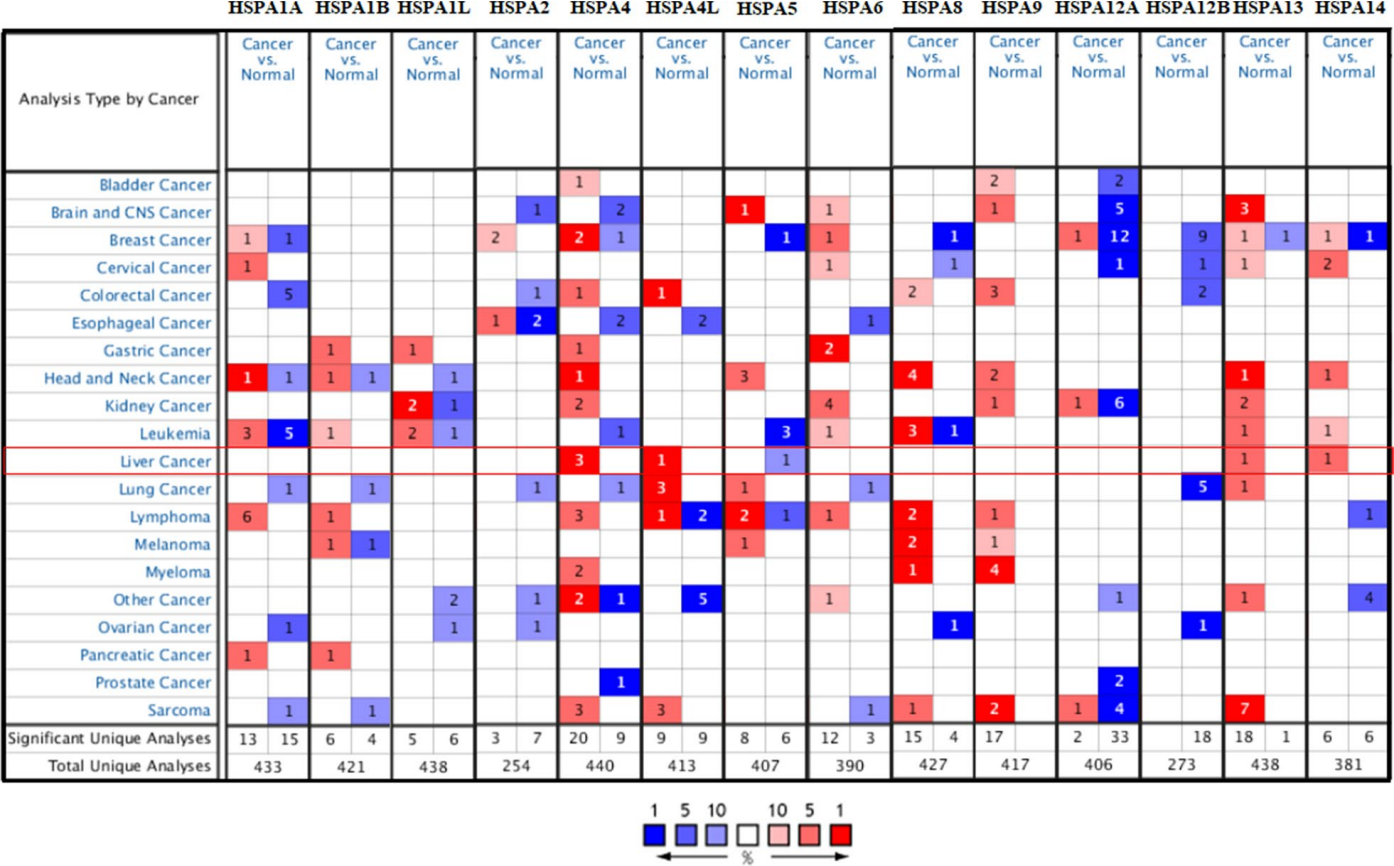
The transcriptional level of HSP70s in Oncomine dataset of different types of cancer

**Fig. 2:**
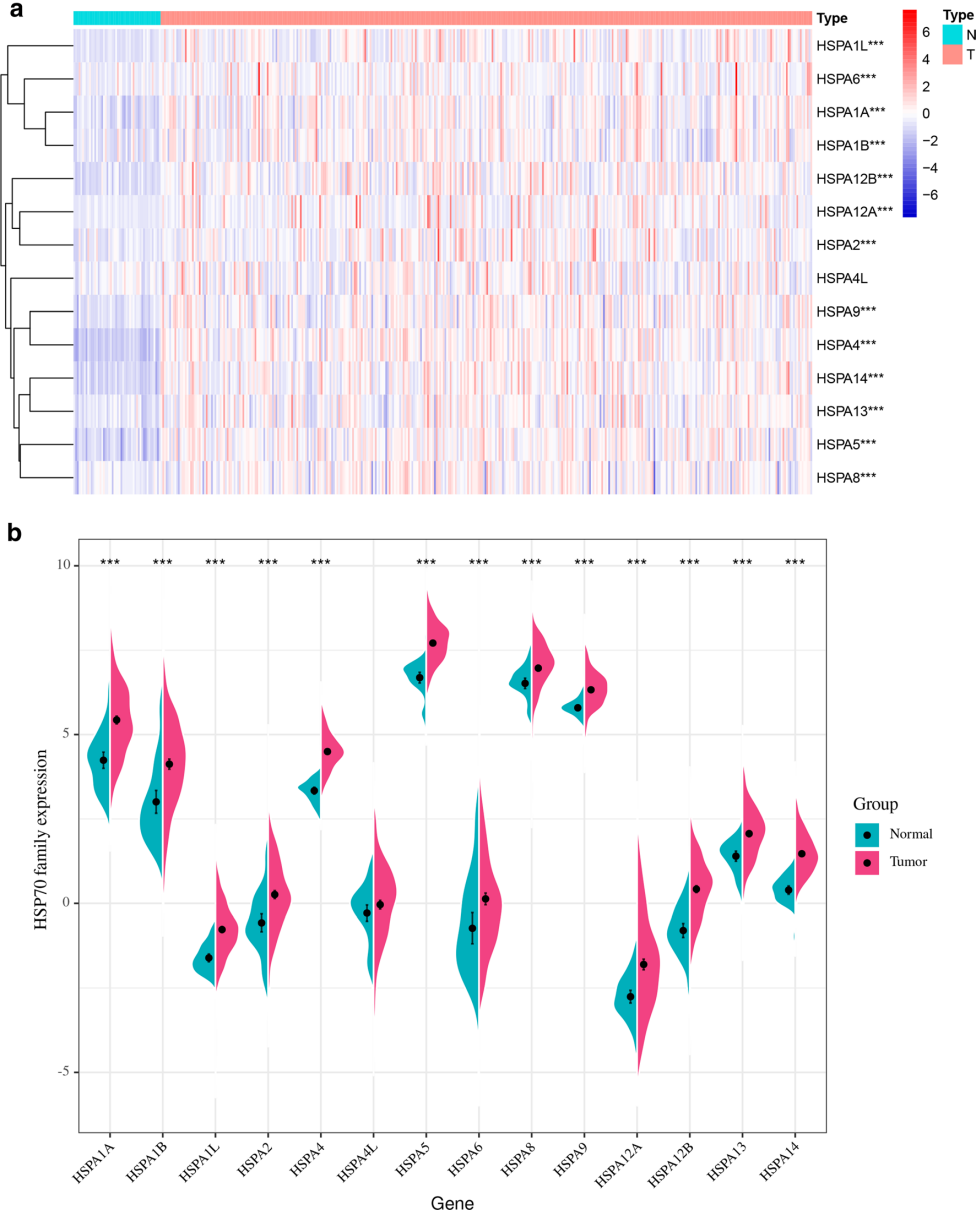
HSP70s is differentially expressed between HCC and normal tissues. The mRNA of all HSP70s are higher than that of normal tissues (p < 0.05)

ONCOMINE and TCGA databases were used to analyze the mRNA expression of HSP70s to explore the different prognosis and potential therapeutic value of HSP70s in HCC patients. ONCOMINE database datasets showed that the mRNA expression of HSPA4/4L/13/14 was significantly higher in liver cancer tissues. Roessler Liver 2 dataset showed significant HSPA4/13/14 overexpression in HCC tissues compared with the normal tissues with multiple changes of 2.202 times (p = 1.9E−72), 2.119 times (p = 9.4E−49), and 2.099 times (p = 7.76E−58), respectively. Wurmbach dataset observed that the mRNA expressions of HSPA4 and HSPA4L increased 2.053 times (p = 3.49E−06) and 5.204 times (p = 5.26E−07), respectively in HCC samples. Roessler dataset revealed that the mRNA expression of HSPA4 increased 2.492 times in HCC tissues (p = 1.21E−08) (Fig. 1; Table 2). The expression profile data of LIHC patients in the TCGA database were also analyzed. The mRNA expression level of HSP70s was significantly upregulated in HCC tissues (all p < 0.05) (Fig. 2).

**Table 2:** There was a significant change in the expression of HSP70s at the transcriptional level of (ONCOMINE) between hepatocellular carcinoma and normal liver tissues

	Types of HCC VS. Liver	Fold change	P value	t-test	Ref
HSPA4	Hepatocellular Carcinoma	2.202	1.90E−72	22.549	Roessler Liver 2
	Hepatocellular Carcinoma	2.492	1.21E−08	7.023	Roessler Liver
	Hepatocellular Carcinoma	2.053	3.49E−06	5.635	Wurmbach Liver
HSPA4L	Hepatocellular Carcinoma	5.204	5.26E−07	7.269	Wurmbach Liver
HSPA13	Hepatocellular Carcinoma	2.119	9.40E−49	17.614	Roessler Liver 2
HSPA14	Hepatocellular Carcinoma	2.099	7.76E−58	19.057	Roessler Liver 2

### Prognostic value of differentially expressed HSP70s in hepatocellular carcinoma patients

**Fig. 3:**
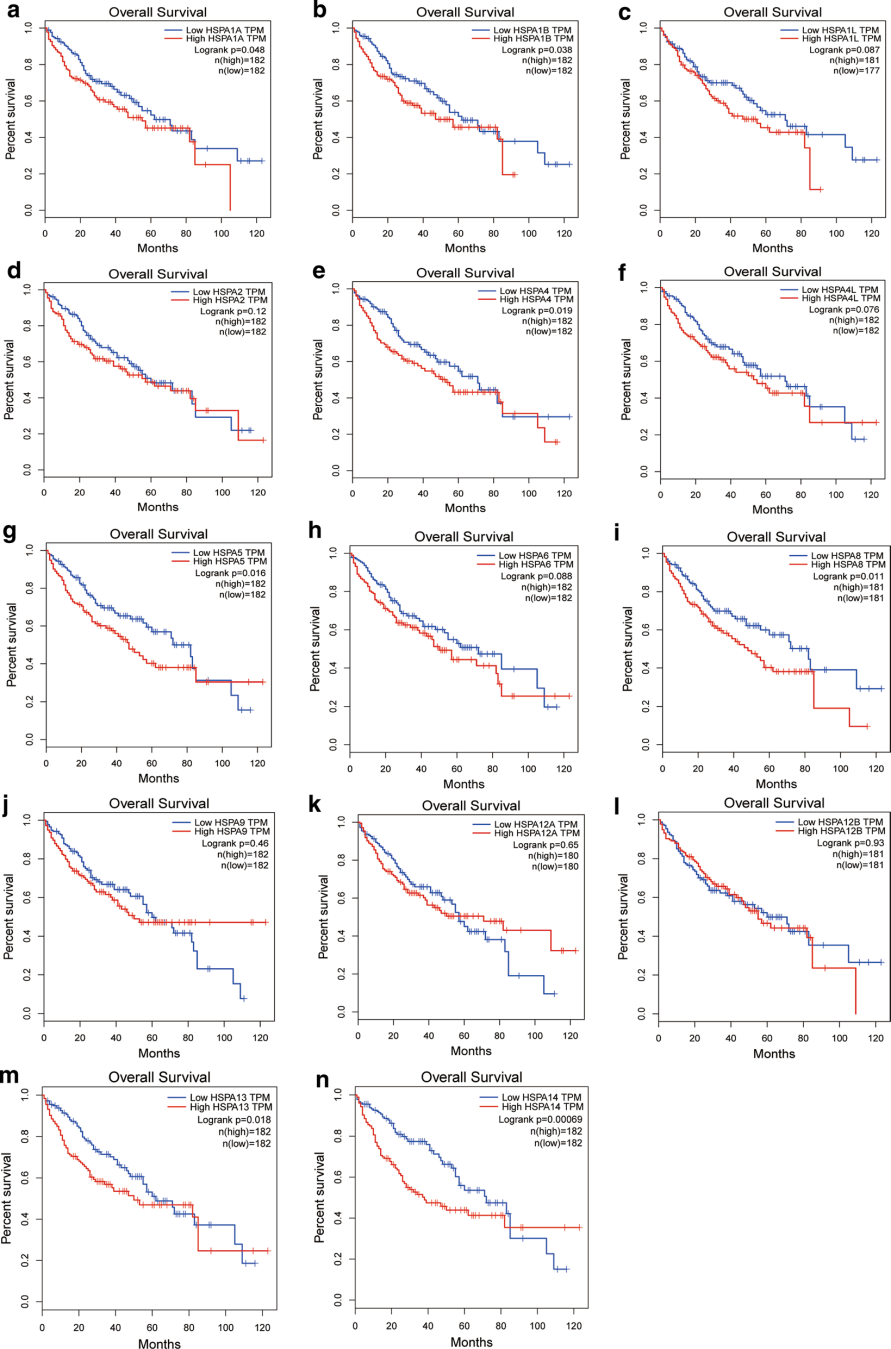
Prognostic value of different HSP70s mRNA expression in hepatocellular carcinoma. The mRNA levels of HSPA1A (**A**), HSPA1B (**B**), HSPA4 (**E**), HSPA5 (**G**), HSPA8 (**I**), HSPA13 (**M**), HSPA14 (**N**) were negatively correlated with the shorter OS in patients with HCC. However, the expression levels of HSPA1L (**C**), HSPA2 (**D**), HSPA4L (**F**), HSPA6 (**H**), HSPA9 (**J**), HSPA12A (**K**) and HSPA12B (**L**) mRNA were not related to the prognosis of patients with HCC (p > 0.05)

The correlation between HSP70 expressions in HCC patients and the overall survival of HCC patients in the TCGA database was further analyzed. The mRNA expression of nearly half of HSP70s was associated with the prognosis of HCC patients. HSPA1A (p = 0.048), HSPA1B (p = 0.038), HSPA4 (p = 0.019), HSPA5 (p = 0.016), HSPA8 (p = 0.011), HSPA13 (p = 0.018) and HSPA14 (p = 0.00069)were associated with shorter OS. However, HSPA1L, HSPA2, HSPA4L, HSPA6, HSPA9, HSPA12A, and HSPA12B mRNA expressions were not associated with the prognosis of HCCC patients (Fig. 3). Therefore, the expression levels of HSPA1A, HSPA1B, HSPA4, HSPA5, HSPA8, HSPA13, and HSPA14 are significantly associated with the prognosis of HCC patients and can be used as potential biomarkers to predict prognosis.

### Relationship between HSP70 expressions and clinicopathological parameters in HCC patients

**Fig. 4:**
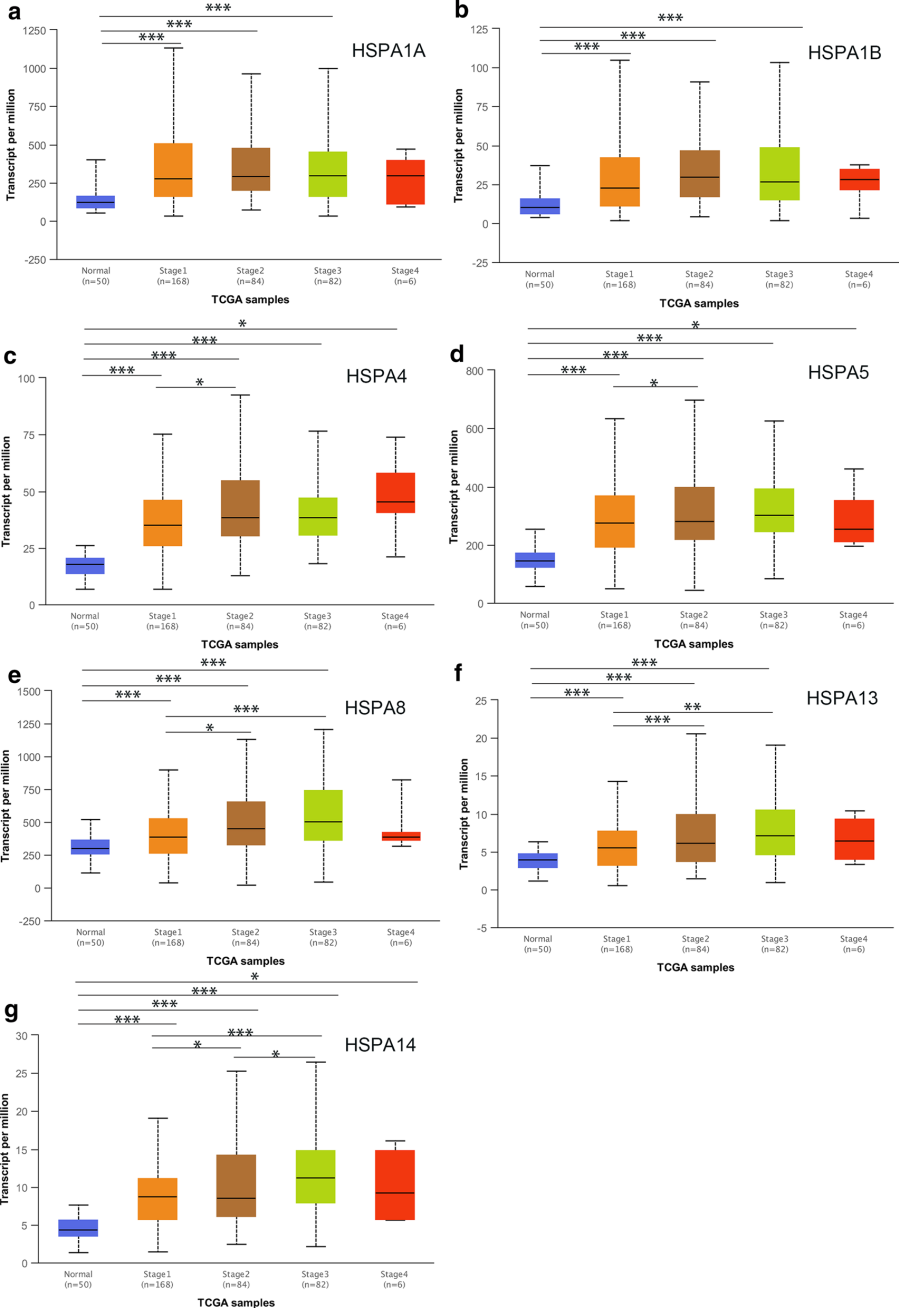
The relationship between the level of 7 HSP70s and neoplasm histologic grade of patients with HCC. With the increase of mRNA expression, their tumor grade tended to be higher. HSPA4 and HSPA14 were most significant among different histologic grade

**Fig. 5:**
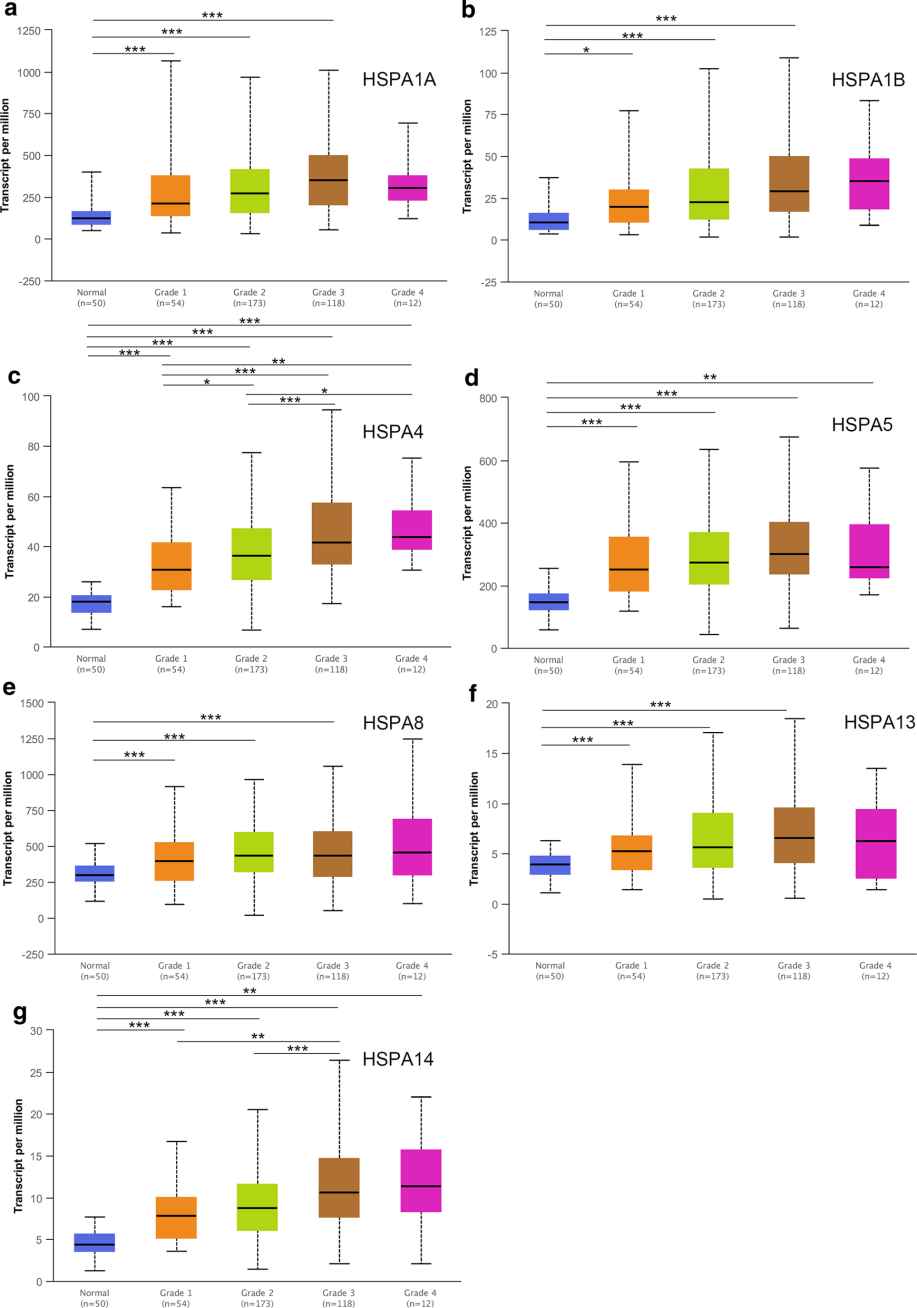
The relationship between the levels of 7 HSP70s and neoplasm disease stage of patients with HCC. With the increase of the mRNA expression, their tumor grade tended to be higher. HSPA4 and HSPA14 were most significant among different disease stage

The relationship between HSP70expressions (novel prognostic markers) and clinicopathological parameters of HCC patients, including individual tumor grade and cancer stage, was further analyzed. The expression of seven HSP70s with prognostic value was significantly associated with the individual cancer stage and tumor grade. Furthermore, the HSP70 mRNA expression levels were higher at the high cancer stage (Fig. 4). The tumor grade increased with the increased mRNA expression. HSPA4 and HSPA14 expressions were significantly associated with various tumor grades (Fig. 5). Therefore, the mRNA expression of seven HSP70s with prognostic value is positively correlated with clinicopathological parameters.

### HSP70s correlation analysis and gene mutation

**Fig. 6:**
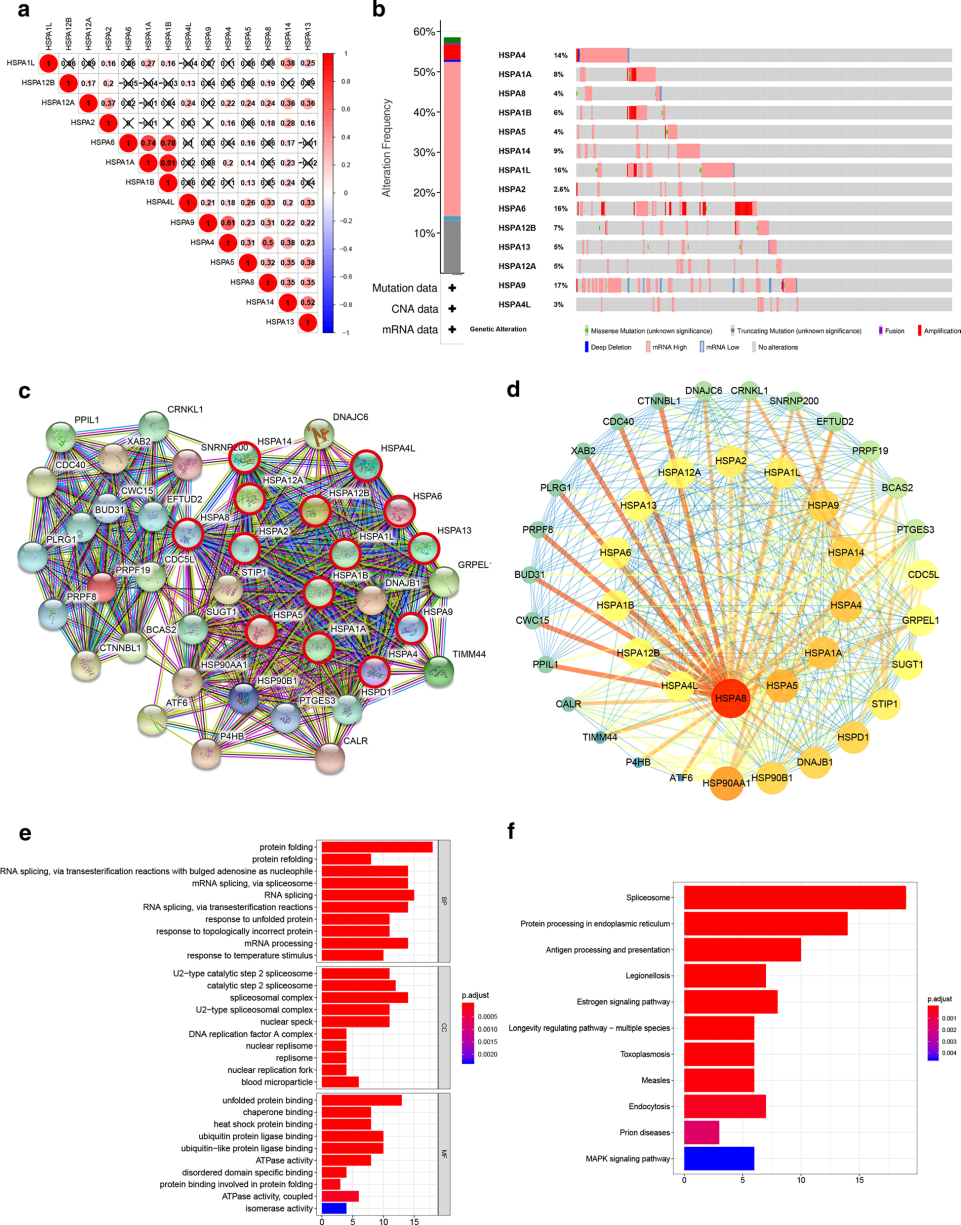
Analysis of correlation among HSP70s, gene mutation and functional enrichment analysis of HSP70. **A** HSPA1A and HSPA1B, HSPA4 and HSPA8, HSPA4 and HSPA9, HSPA6 and HSPA1A, HSPA6 and HSPA1B, HSPA13 and HSPA14 had very significant correlation (correlation coefficient > 0.3). **B** The mutation rate of HSP70s gene was 58.55%. the mutation rates of HSPA1L, HSPA4, HSPA6 and HSPA9 are all higher than 10%, mRNA High and Amplification are the most common mutation types. **C **Thirty neighbor genes significantly related to HSP70s were found through STRING database. **D **Using Cytoscape 3.7.1 to display PPI results through inter-gene connectivity, we found that HSPA8 and HSPA5 played a central role in this network. Then, through the analysis, annotation, visualization and comprehensive discovery of GO (**E**) and KEGG (**F**) analysis in DAVID database

The interaction between 14 HSP70s was also analyzed by assessing HSP70expressions to understand their relationship. HSPA1A and HSPA1B, HSPA4 and HSPA8, HSPA4 and HSPA9, HSPA6 and HSPA1A, HSPA6 and HSPA1B, HSPA13 and HSPA14 were significantly correlated (Fig. 6A). The genetic variation of HSP70s was then analyzed. HCC patients had a high mutation rate of HSP70s. A total of 202 of the 345 sequenced HCC patients showed genetic variations (58.55%). The mutation rates of HSPA1L, HSPA4, HSPA6 and HSPA9 were more than 10%. High and Multiple Alterations were the most common mutations (Fig. 6B).

### Construction of an interaction network of HSP70s and 30 related genes and prediction of their functions and pathways

HSP70 correlation analysis showed that 30 neighbor genes were significantly related to HSP70s in the STRING database (Fig. 6C). Cytoscape 3.7.1 was used to visualize the PPI results via the inter-gene connectivity (Fig. 6D). HSPA8 and HSPA5 played a central part in this network, and the HSP90 and HSP70 families were closely related. Analysis, annotation, visualization, and comprehensive discovery of GO and KEGG enrichment in the DAVID database were used to predict the function of HSP70s and explore 30 adjacent genes. The 30 genes were significantly related to HSP70s and their potential molecular mechanisms. “Protein folding”, “U2-type catalytic step2 spliceosome” and “Unfolded Protein binding” are the most common biological terms in biological processes, molecular functions, and cellular components. Most genes were upregulated in Spliceosome and Protein processing pathways in the endoplasmic reticulum (Fig. 6F).

### Independent prognostic value of HSP70 expressions in HCC patients

**Fig. 7:**
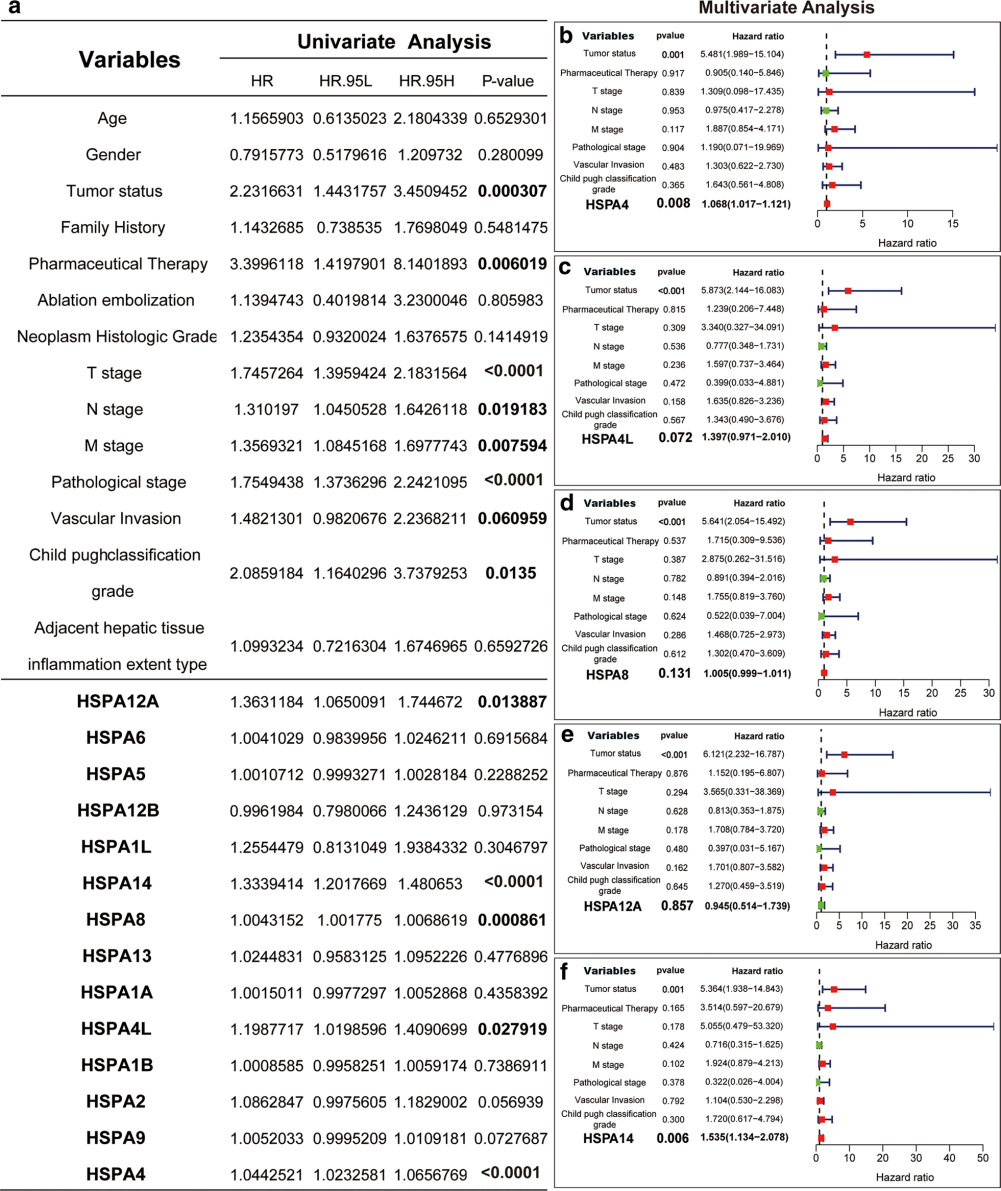
Univariate and multivariate Cox regression analyses of the association between clinicopathological factors (including HSP70s) and overall survival of patients

Independent prognostic value evaluation of HSP70 mRNA expression and OS in HCC patients showed HSPA1A, HSPA1B, HSPA4, HSPA5, HSPA8, HSPA13, and HSPA14 were significantly associated with HCC prognosis. Univariate analysis indicated that the TNM stage, Pathological stage, Child pugh classification grade with higher Tumor status, Pharmaceutical Therapy were associated with shorter OS in HCC patients. The higher mRNA expression of HSPA4L, HSPA4, HSPA8, HSPA12A, and HSPA14 was associated with shorter OS in HCC patients (Fig. 7A). Further multivariate analysis revealed that the higher mRNA expression of HSPA4 and HSPA14 was associated with shorter OS in HCC patients (Fig. 7B). Therefore, the expression levels of HSPA4 and HSPA14 are independent prognostic factors of OS in HCC patients.

### Interfering with the expression of HSPA4 or HSPA14 inhibits the proliferation, invasion and metastasis of hepatoma cells

**Fig. 8:**
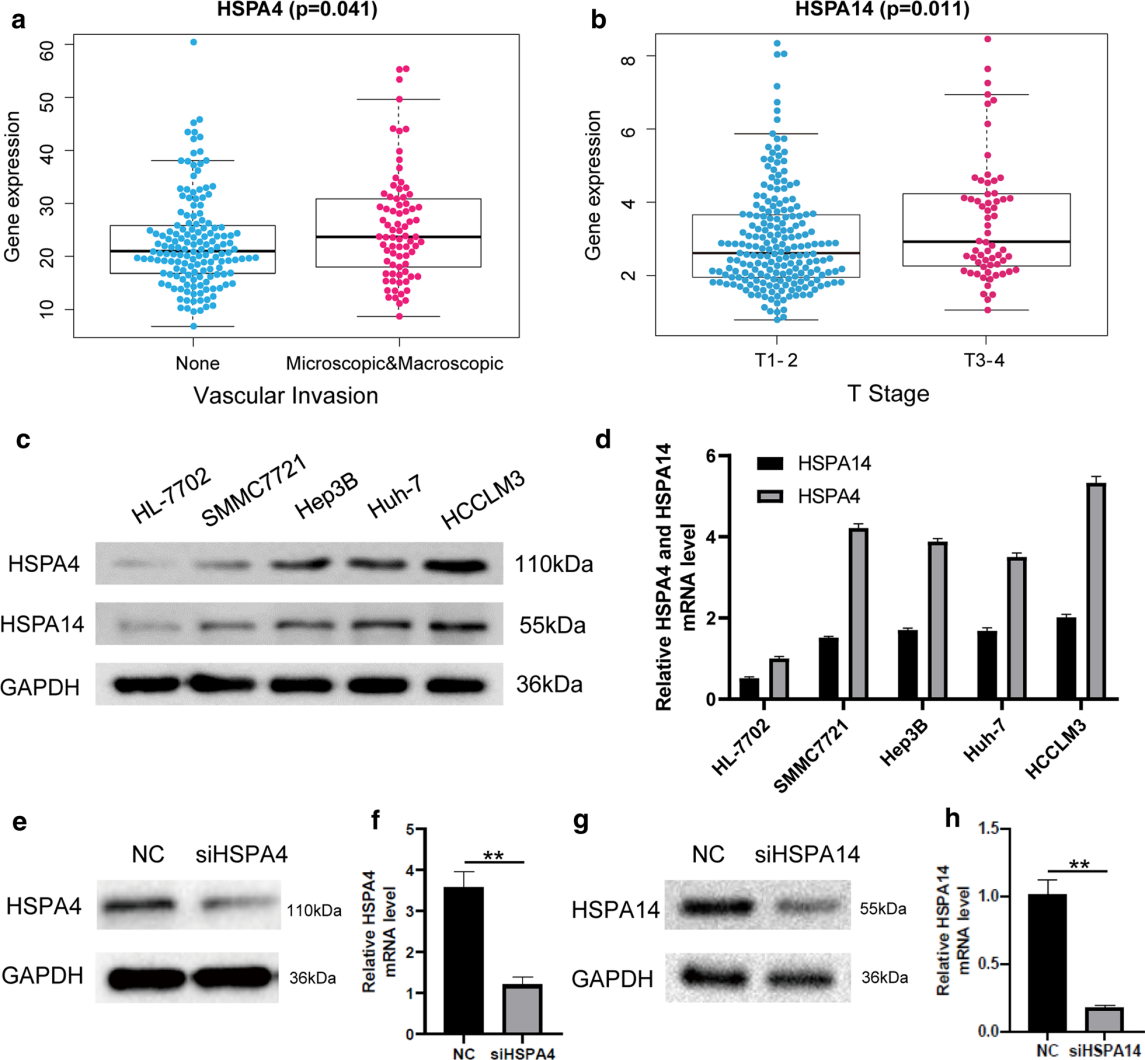
Expression of HSPA4 and HSPA14 in normal liver cells and HCC cells. **A** The expression of HSPA4 was positively correlated with vascular invasion. **B** The expression level of HSPA14 was positively correlated with higher T stage. **C **and **D** The results of Western Blot and qPCR revealed that HCCLM3 cells demonstrate relatively high levels of HSPA4 and HSPA14 expression. **E** and** F** The expression of HSPA4 in siHSPA4 is less than that in NC group, and the expression of HSPA14 was less than that in NC group(p < 0.01), the NC group was the negative control group

**Fig. 9:**
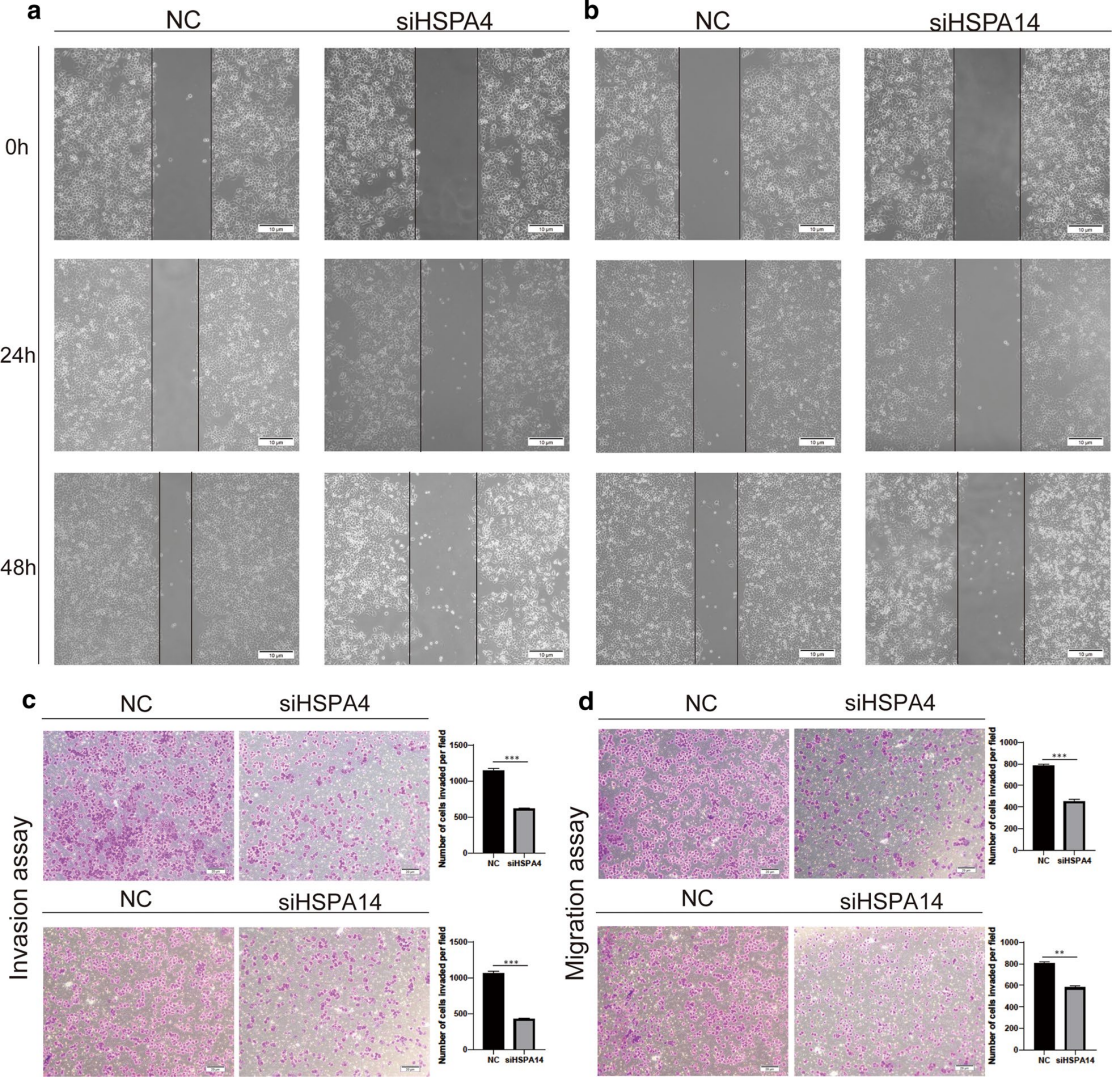
Knockdown of HSPA4 and HSPA14 expression inhibits HCC invasion and metastasis in vitro. The distance of scratche in siHSPA4 group is no significant change but that in NC group decreased (**A**). while that in siHSPA14 group is no significant change, but in NC group decreased (**B**). The number of cells in siHSPA4 group (**C**) and siHSPA14 group (**D**) were significantly less than that in NC group in invasion and migration assay (p < 0.01), the NC group was the negative control group

**Fig. 10:**
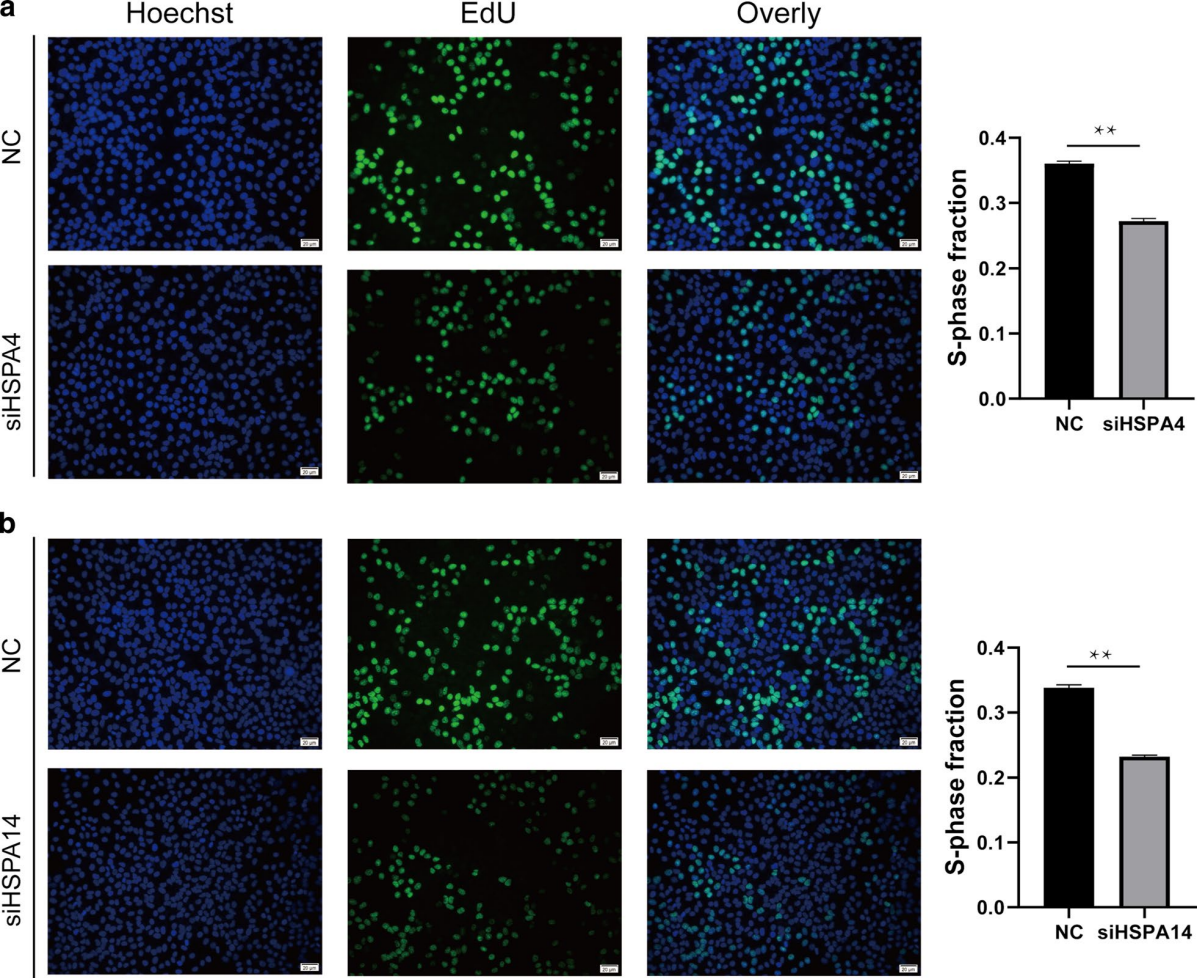
Knockdown of HSPA4 and HSPA14 expression inhibits HCC proliferation in vitro. The number of proliferative cells in siHSPA4 group was less than that in NC group (**A**); The number of proliferative cells in siHSPA14 group was less than that in NC group (**B**), the NC group was the negative control group

The high HSPA4 expression was associated with vascular invasion, while the high HSPA14 expression with the higher T stage of the tumor (Fig. 8A and B). Therefore, the high HSPA4 and HSPA14 expressions are associated with tumor proliferation, invasion and metastasis, indicating that their high expressions could influence the prognosis of HCC patients by accelerating tumor proliferation, invasion, and metastasis of tumor. Cell function experiments were used to confirm the conjecture. Western Blot and qPCR were used to evaluate HSPA4 and HSPA14 expressions in normal liver cells (HL-7702) and HCC cells. The results revealed that HCCLM3 cells demonstrate relatively high levels of HSPA4 and HSPA14 expression (Fig. 8C and D). Therefore, HSPA4 and HSPA14 expressions in HCCLM3 cells were knocked down via interference fragments. PCR and Western blotting results revealed that HSPA4 and HSPA14 expressions significantly decreased in HCCLM3 cells (Fig. 8E–H). The NC group was the negative control group. A scratch experiment was then conducted using HCCLM3 cells. The scratch spacing significantly decreased in the NC group after 48 h, but there was no significant change in HSPA4 and HSPA14 knockdown groups, indicating that the migration ability of hepatoma cells decreased (Fig. 9A and B), similar to the migration and invasion assays. (Fig. 9C and D) Besides, Edu proliferation assays suggested that cell proliferation ability was significantly decreased after HSPA4 and HSPA14 expression interferences(Fig. 10A and B).

## Discussion

HCC is a serious malignant tumor in the world due to its complex molecular and cellular heterogeneity. Besides, HCC incidence continues to increase [32]. Over 200 genes related to HCC proliferation, invasion and metastasis have been reported. However, the specific prognostic biomarkers and therapeutic targets are insufficient [33]. Therefore, the screening of HCC molecular biological markers could improve prognosis and reduce mortality.

HSP70s are conserved and inducible heat shock proteins essential in protein homeostasis [34]. HSP70s inhibit non-specific protein aggregation and help proteins to obtain normal structure. HSP70s also have anti-apoptotic properties and are involved in many processes such as tumor cell proliferation, invasion, metastasis, and death [35]. HSP70s helps cells maintain protein homeostasis. The functional enrichment analysis showed that HSP70s are mainly involved in protein folding and unfolded protein reactions. Related HCC research showed that some HSP70s play a crucial role. However, it is necessary to determine the several roles of HSP70s in HCC. This is the first study to systematically investigate the expression, mutation, and prognostic value of various HSP70s in HCC.

Bioinformatics analysis indicated that 14 HSP70s are significantly increased in HCC. The high expression levels of HSPA1A, HSPA1B, HSPA4, HSPA5, HSPA8, HSPA13, and HSPA14 were significantly negatively correlated with overall survival. However, their high expression was positively associated with higher tumor grade and cancer stage. Some studies have shown that HSPA1A can enhance the apoptosis resistance of H22 cells to promote tumor growth [12] by activating NF- κB, and HSPA1A of extracellular to the endogenous ligand of TLR4 and TLR2 [12]. Garg et al. [36] did IHC analysis and RT-PCR and showed that HSPA1B (HSP70-2) is overexpressed in 86% of tumor tissues. Also, down-regulated HSP70-2 expression substantially inhibited cell growth, colony formation, migration, and invasion in vitro and inhibited tumor growth in vivo. Nirmala Jagadish et al. [14] also used RT-PCR and IHC to detect the high HSP 70-2 expressions in 78% of CRC patients. The decreased HSP 70-2 expression causes growth inhibition, colony formation, migration, and CRC cells invasion. Moreover, reduced HSP 70-2 expressions significantly decreased the tumor growth in the COLO205 human transplanted tumor model in mice. Little research has been conducted on the relationship between HSPA13 (STcH) and tumors. Zongguo Yang et al. [37] indicated that HSPA13 is overexpressed in tumor tissues. While HSPA1A, HSPA1B, and HSPA13 were negatively correlated with the overall survival of patients, they were highly expressed in HCC. The higher HSPA1A, HSPA1B, and HSPA13 expressions were positively correlated with higher cancer stage and tumor grade.

In this study, HSPA5 and HSPA8 were highly expressed in HCC, and their high expression was negatively associated with the overall survival of patients. HSPA5 and HSPA8 were in the center of the PPI network, suggesting that they are involved in the occurrence and development of hepatocellular carcinoma via several mechanisms. Therefore, HSPA5 and HSPA8 can be prognostic biomarkers in HCC diagnosis and treatment. A previous study showed that HSPA5 regulates FAT10 expression by modulating the NF- κ B pathway and directly activating the NF- κB pathway. Besides, the study showed that FAT10 is an anti-tumor suppressor p53 gene [17], and HSPA5 regulates HCC invasion and metastasis via the HSPA5-LRP6-HOXB9 axis [18]. Some studies have shown that HSPA8 (HSC 70, HSP 71, HSP 71, or HSP 73) is overexpressed in endometrial carcinoma. siRNA of HSPA8 transfection into endometrial cancer cells significantly inhibited cell proliferation and growth and promoted apoptosis. HSPA8 is key in the occurrence and development of endometrial carcinoma [21]. The above studies have shown that HSP70s promote the occurrence and development of tumor cells, and their high expression is associated with the poor prognosis of tumor patients.

Finally, whether HSP70s can be used as an independent risk factor to predict the prognosis of hepatocellular carcinoma patients was analyzed. Univariate and multivariate analyses showed that could predict the prognosis of HCC patients. High HSPA4 and HSPA14 expressions were also associated with vascular infiltration and higher T stage in HCC patients. HSPA4 and HSPA14 expression interference showed inhibited Cell proliferation, invasion, and metastasis. Zongguo Yang et al. [37] analyzed HSPA4 expressions in 220 HCC patients and found that HSPA4 is overexpressed in tumor tissues. They also showed that HSPA4 is associated with early HCC recurrence. HSPA4 overexpression is associated with the poor prognosis of breast cancer patients [19]. HSPA4 is a potential cancer stem cell (CSC) marker for gastric cancer [38]. While some studies have shown that HSPA14 (HSP70L1) is associated with the overall survival and recurrence of cervical cancer [39], siRNA-mediated inhibition of HSPA14 can reduce the migration, invasion, and transformation activity of NBS-1 overexpressed cells [40]. However, the mechanism of HSPA4 and HSPA14 effect on the occurrence and development of hepatocellular carcinoma is unknown and should be studied.

In conclusion, the differential expression of HSP70s in HCC and its potential diagnostic and prognostic value showed that HSP70s are highly expressed in HCC and are associated with poor prognosis. Further analysis showed that HSPA1A, HSPA1B, HSPA4, HSPA5, HSPA8, HSPA13, and HSPA14 could be used as prognosis biomarkers, and HSPA4 HSPA14 as independent risk factors to determine the prognosis of HCC patients. Besides, knockdowned HSPA4 or HSPA14 can inhibit HCC proliferation, invasion, and metastasis, indicating HSP70s promote HCC occurrence and development. Moreover, HSPA4 and HSPA14 can be novel biomarkers and therapeutic targets for HCC patients.

## Supplementary Information

**Additional file 1: Table S1.** The specific primers used for PCR amplification.

## Data Availability

The transcriptome and clinicopathological data set were obtained from TCGA (https://www.cancer.gov/tcga).

## References

[1] Bray F, Ferlay J, Soerjomataram I, Siegel RL, Torre LA, Jemal A (2018). Global cancer statistics 2018: GLOBOCAN estimates of incidence and mortality worldwide for 36 cancers in 185 countries. CA Cancer J Clin.

[2] Ingle PV, Samsudin SZ, Chan PQ, Ng MK, Heng LX, Yap SC, Chai AS, Wong AS (2016). Development and novel therapeutics in hepatocellular carcinoma: a review. Ther Clin Risk Manag.

[3] Rosenzweig R, Nillegoda NB, Mayer MP, Bukau B (2019). The Hsp70 chaperone network. Nat Rev Mol Cell Biol.

[4] Calderwood SK, Gong J (2016). Heat shock proteins promote cancer: it’s a protection racket. Trends Biochem Sci.

[5] Wang Z, Gou W, Liu M, Sang W, Chu H, Zhang W (2015). Expression of P53 and HSP70 in chronic hepatitis, liver cirrhosis, and early and advanced hepatocellular carcinoma tissues and their diagnostic value in hepatocellular carcinoma: an immunohistochemical study. Med Sci Monit.

[6] Miao W, Fan M, Huang M, Li JJ, Wang Y (2019). Targeted profiling of heat shock proteome in radioresistant breast cancer cells. Chem Res Toxicol.

[7] Giri B, Sethi V, Modi S, Garg B, Banerjee S, Saluja A, Dudeja V (2017). "Heat shock protein 70 in pancreatic diseases: friend or foe". J Surg Oncol.

[8] Moradi-Marjaneh R, Paseban M, Moradi Marjaneh M (2019). Hsp70 inhibitors: implications for the treatment of colorectal cancer. IUBMB Life.

[9] Kumar S, Gurshaney S, Adagunodo Y, Gage E, Qadri S, Sharma M, Malik S, Manne U, Singh U, Singh R (2018). Hsp70 and gama-Semino protein as possible prognostic marker of prostate cancer. Front Bioscience (Landmark edition).

[10] Cho W, Jin X, Pang J, Wang Y, Mivechi NF, Moskophidis D (2019). The molecular chaperone heat shock protein 70 controls liver cancer initiation and progression by regulating adaptive DNA damage and mitogen-activated protein kinase/extracellular signal-regulated kinase signaling pathways. Mol Cell Biol..

[11] Xiao H, Wang B, Xiong HX, Guan JF, Wang J, Tan T, Lin K, Zou SB, Hu ZG, Wang K (2021). A novel prognostic index of hepatocellular carcinoma based on immunogenomic landscape analysis. J Cell Physiol.

[12] Wu FH, Yuan Y, Li D, Liao SJ, Yan B, Wei JJ, Zhou YH, Zhu JH, Zhang GM, Feng ZH (2012). Extracellular HSPA1A promotes the growth of hepatocarcinoma by augmenting tumor cell proliferation and apoptosis-resistance. Cancer Lett.

[13] Lee JH, Han YS, Yoon YM, Yun CW, Yun SP, Kim SM, Kwon HY, Jeong D, Baek MJ, Lee HJ (2017). Role of HSPA1L as a cellular prion protein stabilizer in tumor progression via HIF-1alpha/GP78 axis. Oncogene.

[14] Jagadish N, Parashar D, Gupta N, Agarwal S, Suri V, Kumar R, Suri V, Sadasukhi TC, Gupta A, Ansari AS (2016). Heat shock protein 70 – 2 (HSP70-2) is a novel therapeutic target for colorectal cancer and is associated with tumor growth. BMC Cancer.

[15] Zhai LL, Xie Q, Zhou CH, Huang DW, Tang ZG, Ju TF (2017). Overexpressed HSPA2 correlates with tumor angiogenesis and unfavorable prognosis in pancreatic carcinoma. Pancreatology.

[16] Zhang H, Gao H, Liu C, Kong Y, Wang C, Zhang H (2015). Expression and clinical significance of HSPA2 in pancreatic ductal adenocarcinoma. Diagn Pathol.

[17] Luo C, Xiong H, Chen L, Liu X, Zou S, Guan J, Wang K (2018). GRP78 Promotes Hepatocellular Carcinoma proliferation by increasing FAT10 expression through the NF-kappaB pathway. Exp Cell Res.

[18] Xiong H, Xiao H, Luo C, Chen L, Liu X, Hu Z, Zou S, Guan J, Yang D, Wang K (2019). GRP78 activates the Wnt/HOXB9 pathway to promote invasion and metastasis of hepatocellular carcinoma by chaperoning LRP6. Exp Cell Res.

[19] Gu Y, Liu Y, Fu L, Zhai L, Zhu J, Han Y, Jiang Y, Zhang Y, Zhang P, Jiang Z (2019). Tumor-educated B cells selectively promote breast cancer lymph node metastasis by HSPA4-targeting IgG. Nat Med.

[20] Khosla R, Hemati H, Rastogi A, Ramakrishna G, Sarin SK, Trehanpati N (2019). miR-26b-5p helps in EpCAM + cancer stem cells maintenance via HSC71/HSPA8 and augments malignant features in HCC. Liver Int.

[21] Shan N, Zhou W, Zhang S, Zhang Y (2016). Identification of HSPA8 as a candidate biomarker for endometrial carcinoma by using iTRAQ-based proteomic analysis. Onco Targets Ther.

[22] Starenki D, Hong SK, Lloyd RV, Park JI (2015). Mortalin (GRP75/HSPA9) upregulation promotes survival and proliferation of medullary thyroid carcinoma cells. Oncogene.

[23] Cui X, Li Z, Piao J, Li J, Li L, Lin Z, Jin A (2017). Mortalin expression in pancreatic cancer and its clinical and prognostic significance. Hum Pathol.

[24] Ma H, Lu T, Zhang X, Li C, Xiong J, Huang L, Liu P, Li Y, Liu L, Ding Z (2015). HSPA12B: a novel facilitator of lung tumor growth. Oncotarget.

[25] Chen W, Liu X, Yuan S, Qiao T (2018). HSPA12B overexpression induces cisplatin resistance in non-small-cell lung cancer by regulating the PI3K/Akt/NF-kappaB signaling pathway. Oncol Lett.

[26] Rhodes D, Yu J, Shanker K, Deshpande N, Varambally R, Ghosh D, Barrette T, Pandey A, Chinnaiyan A (2004). ONCOMINE: a cancer microarray database and integrated data-mining platform. Neoplasia (New York NY).

[27] Chandrashekar D, Bashel B, Balasubramanya S, Creighton C, Ponce-Rodriguez I, Chakravarthi B, Varambally S (2017). UALCAN: a portal for facilitating tumor subgroup gene expression and survival analyses. Neoplasia..

[28] Gao J, Aksoy B, Dogrusoz U, Dresdner G, Gross B, Sumer S, Sun Y, Jacobsen A, Sinha R, Larsson E (2013). Integrative analysis of complex cancer genomics and clinical profiles using the cBioPortal. Sci Signal.

[29] Tomczak K, Czerwińska P, Wiznerowicz M (2015). The Cancer Genome Atlas (TCGA): an immeasurable source of knowledge. Contemporary oncology (Poznan Poland).

[30] Yuan R, Wang K, Hu J, Yan C, Li M, Yu X, Liu X, Lei J, Guo W, Wu L (2014). Ubiquitin-like protein FAT10 promotes the invasion and metastasis of hepatocellular carcinoma by modifying beta-catenin degradation. Cancer Res.

[31] Liu X, Chen L, Ge J, Yan C, Huang Z, Hu J, Wen C, Li M, Huang D, Qiu Y (2016). The ubiquitin-like protein FAT10 stabilizes eEF1A1 expression to promote tumor proliferation in a complex manner. Cancer Res.

[32] Bruix J, Gores GJ, Mazzaferro V (2014). Hepatocellular carcinoma: clinical frontiers and perspectives. Gut.

[33] Craig AJ, Villanueva A (2016). Liver capsule: Molecular-based signatures in hepatocellular carcinoma. Hepatology.

[34] Elmallah MIY, Cordonnier M, Vautrot V, Chanteloup G, Garrido C, Gobbo J (2020). Membrane-anchored heat-shock protein 70 (Hsp70) in cancer. Cancer Lett.

[35] Lianos GD, Alexiou GA, Mangano A, Mangano A, Rausei S, Boni L, Dionigi G, Roukos DH (2015). The role of heat shock proteins in cancer. Cancer Lett.

[36] Garg M, Kanojia D, Saini S, Suri S, Gupta A, Surolia A, Suri A (2010). Germ cell-specific heat shock protein 70 – 2 is expressed in cervical carcinoma and is involved in the growth, migration, and invasion of cervical cells. Cancer.

[37] Yang Z, Zhuang L, Szatmary P, Wen L, Sun H, Lu Y, Xu Q, Chen X (2015). Upregulation of heat shock proteins (HSPA12A, HSP90B1, HSPA4, HSPA5 and HSPA6) in tumour tissues is associated with poor outcomes from HBV-related early-stage hepatocellular carcinoma. Int J Med Sci.

[38] Morisaki T, Yashiro M, Kakehashi A, Inagaki A, Kinoshita H, Fukuoka T, Kasashima H, Masuda G, Sakurai K, Kubo N (2014). Comparative proteomics analysis of gastric cancer stem cells. PLoS One.

[39] Hu YX, Zheng MJ, Zhang WC, Li X, Gou R, Nie X, Liu Q, Hao YY, Liu JJ, Lin B (2019). Systematic profiling of alternative splicing signature reveals prognostic predictor for cervical cancer. J Transl Med.

[40] Yang MH, Chiang WC, Chou TY, Chang SY, Chen PM, Teng SC, Wu KJ (2006). Increased NBS1 expression is a marker of aggressive head and neck cancer and overexpression of NBS1 contributes to transformation. Clin Cancer Res.

