# Supplementary material for: The expression profiles and prognostic values of HSP70s in hepatocellular carcinoma
Source: Cancer Cell Int. 2021 May 31;21:286. doi: 10.1186/s12935-021-01987-9 (PMC8165812; doi:10.1186/s12935-021-01987-9)
Supplement: Supplementary file 1 — Additional file 1: Table S1. The specific primers used for PCR amplification. [file 12935_2021_1987_MOESM1_ESM.docx]

Table S1(The specific primers used for PCR amplification)

| Primer Name | Sequence(5＇to 3＇) |
| --- | --- |
| HSPA14-F | TTGCAAATGATGCCGGTGAC |
| HSPA14-R | CTGAGCTTGTGGATCACTGGA |
| HSPA4-F | GACATGGGCCACTCTGCTTA |
| HSPA4-R | TGCAGTGGCCAGAACTTTCA |
| GAPDH-F | CTTCATTGACCTCAAGACA |
| GAPDH-R | ACTCCACGACATACTCAGC |
